# The Role of MEF2 in Scar Formation and Angiogenesis

**DOI:** 10.1111/jocd.70769

**Published:** 2026-03-12

**Authors:** Rui Tao, Yajuan Song, Zhou Yu, Yankun Guo, Yuye Cao, Tong Wang, Peng Guo, Yue Yin

**Affiliations:** ^1^ Department of Plastic Surgery, Xijing Hospital Fourth Military Medical University Xi'an China

**Keywords:** fibroblasts, inflammation, MEF2, scar, transcription factors, vessel, wound healing

## Abstract

**Background:**

Myocyte enhancer factor 2 (MEF2), belonging to the Minichromosome Maintenance 1, Agamous, Deficiens and Serum Response Factor (MADS) box family with four members (MEF2A‐D), plays a pivotal role in the proliferation and differentiation of various cells, including endothelial cells (ECs) and fibroblasts, as well as in physiological processes such as angiogenesis. Recent research highlights the crucial importance of MEF2 in the formation of hypertrophic scar, indicating its potential significance in scar formation and angiogenesis. However, the underlying mechanisms are still largely unexplored and warrant further investigation.

**Aims:**

This article aims to provide a detailed summary of the connections between MEF2 and both angiogenesis and scar formation, with a focus on elucidating the possible mechanisms by which MEF2 participates in angiogenesis and scar formation.

**Methods:**

Search on the Pubmed using the keywords MEF2, Scar, and Angiogenesis. The retrieval period spanned from January to August 2025. Summarize the viewpoints of the articles and refine the mechanisms and interconnections among MEF2, Scar, and Angiogenesis.

**Results:**

Imbalanced regulation of angiogenesis leads to abnormal scar formation. Although the role of MEF2 is not fully understood, it has been hypothesized as follows: MEF2 indirectly influences scar formation by regulating angiogenesis and vascular inflammatory responses, via the MAPK (mitogen‐activated protein kinase) signaling pathway and its interplay with vascular endothelial growth factor (VEGF). On the other hand, MEF2 leads to abnormal deposition of extracellular matrix (ECM) and excessive activation of fibroblasts through its involvement in the transforming growth factor‐β (TGF‐β)/mothers against decapentaplegic (Smad) signaling pathway, ultimately leading to fibrosis and scar formation during the wound healing process.

**Conclusions:**

MEF2 is intimately associated with angiogenesis and scar formation. However, the mechanisms through which MEF2 is involved in these processes remain incompletely understood, necessitating further in‐depth research at the genetic and molecular levels. An increasing number of studies suggest that MEF2 holds significant potential in anti‐angiogenesis and scar treatment therapies, potentially emerging as a novel target for the treatment of pathological scars in the future.

## 1 Introduction

Scar is a necessary outcome of the healing process for most wounds. Compared to ordinary scars, pathological scars exhibit uncontrolled development, are prone to recurrence, and are difficult to treat [1]. Pathological scars not only impair the patient's physical health but also significantly affect their mental health and social functioning due to aesthetic concerns and challenges in treatment [2]. The underlying mechanisms of pathological scar formation are currently not fully understood, and treatment options are limited. Among the various mechanisms involved in pathological scar formation, angiogenesis plays a crucial role. As wound healing progresses to the later stages, pro‐angiogenic factors gradually decline while anti‐angiogenic factors increase. The combined dynamic regulation of these factors results in a gradual decrease in the number of new blood vessels, and existing blood vessels mature and undergo remodeling, allowing the wound to heal normally [3, 4]. If the factors regulating angiogenesis are dysregulated, it can lead to abnormal collagen production and pathological scar formation [5]. Compared to normal tissue, pathological scar tissue exhibits increased angiogenesis [6, 7], and anti‐angiogenic therapy has proven clinically effective in preventing the formation of pathological scars [8]. Therefore, exploring anti‐angiogenic therapy is of great significance for the targeted treatment of pathological scars. Myocyte enhancer factor 2 (MEF2) is a transcription factor belonging to the MADS box family, which was first discovered in relation to muscle tissue and named in 1989 [9]. Later, researchers observed that MEF2 is widely expressed, such as in the cardiovascular system, nervous system, and cancer tissues. An increasing number of studies suggest that MEF2 may play an important role in various regulatory networks and pathophysiological processes related to angiogenesis and scar formation. The relationship between transforming growth factor‐β (TGF‐β)/mothers against decapentaplegic (Smad) signaling pathway and scar formation has been extensively studied.

Histone deacetylase 5 (HDAC5), a member of the class IIa histone deacetylase family, interacts with MEF2 to form an HDAC–MEF2 complex. The epigenetic mechanisms through which HDAC5 modulates chromatin function can be summarized as follows:

- Catalyzing histone deacetylation: HDAC5 removes acetyl groups from lysine residues on histone tails, leading to chromatin condensation and transcriptional repression [10].
- Regulating non‐histone protein acetylation: Beyond histones, HDAC5 can also deacetylate non‐histone proteins, thereby indirectly influencing chromatin structure and gene expression. For instance, inhibition of HDAC5 in hematopoietic stem cells enhances acetylation of p65, facilitating chemokine C‐X‐C receptor‐4 (CXCR4) transcription [10].
- Subcellular localization‐dependent effects: HDAC5 shuttles dynamically between the nucleus and cytoplasm. This trafficking is regulated by specific signaling pathways, such as the AMPK pathway, and influences its access to chromatin targets, thereby modulating gene expression [11].

A recent study has shown that HDAC5 can interfere with the inhibitory effect of Smad7 on the TGF‐β pathway. MEF2 can bind to HDAC5 and inhibit downstream Smad7 in this signaling pathway, ultimately promoting the formation of hypertrophic scar by activating the TGF‐β/Smads signaling pathway [12]. This suggests that MEF2 may have a great impact on angiogenesis and scar formation, with enormous potential in the treatment of scars, and is expected to become a novel target for the treatment of pathological scars in the future. This article will provide a detailed summary of MEF2, angiogenesis, and scar formation, as well as the connection among them, with a focus on elucidating the possible mechanisms by which MEF2 participates in angiogenesis and scar formation.

## 2 Methods

A narrative literature search was conducted using PubMed as the primary database. Key search terms included “MEF2”, “scar”, “angiogenesis”, “fibrosis”, and their combinations, connected by Boolean operators (AND/OR) to refine the results. Given the limited volume of directly relevant publications, the search scope encompassed all articles published between 1989 and 2025. The retrieval period spanned from January to August 2025. Inclusion and exclusion criteria were based on peer‐review status and relevance to the roles of MEF2 in skin scarring and angiogenesis. Study quality was rigorously evaluated during the selection process; only research with high methodological standards, robust design, and scientific rigor was included in this review.

## 3 Brief Introduction of MEF2

MEF2 is a special transcriptional factor that is expressed in various human tissues and organs. MEF2 was found in the skeletal muscle tube nucleus for the first time and was named by Gossett et al. in 1989 [9]. The MEF2 family has four members, including MEF2A, MEF2B, MEF2C, and MEF2D [13]. The N‐terminal region of MEF2 family members exhibits high similarity, featuring downstream MADS‐box and MEF2 domains, and is capable of interacting with DNA, cofactors, and other MEF2 family members; the C‐terminal region does not exhibit high similarity, and it is associated with the transcriptional activation of other MEF2 family members [14]. Earlier research indicated that MEF2 primarily plays a significant role in muscle tissue such as skeletal muscle, myocardium, and smooth muscle. MEF2 can synergistically promote myocyte differentiation and muscle fiber formation with myogenic regulatory factor (MRF). MEF2 also affects muscle regeneration. Combined deletion of MEF2A, MEF2C, and MEF2D can prevent muscle regeneration in mice [15]. However, recent studies have increasingly found that MEF2 is also involved in the physiological and pathological processes of other tissues, such as neural tissue, vascular tissue, and tumor tissue, playing a corresponding role in each (Table 1). MEF2 can play a role in the angiogenesis process and exert an effect on the differentiation, multiplication, and apoptosis of ECs in blood vessels [18]. On the other hand, MEF2 has a significant impact on the physiological activities of neurons, including their development and survival. It limits the number of synapses in neurons and can affect cognitive processes, learning, and memory formation. Furthermore, these effects can even lead to neuropsychiatric diseases [16, 17]. MEF2 plays diverse roles in either promoting or inhibiting tumor cell development across different types of cancer, and even within the same type [19, 20]. Furthermore, MEF2 is also implicated in the skeletal and immune systems, regulating the development and function of bone‐related cells and lymphocytes [21, 22, 23]. Additionally, MEF2 exhibits correlations with angiogenesis, fibrosis, and a variety of diseases (Table 2). Specifically, MEF2A is associated with muscle degeneration, atherosclerosis, among others; MEF2B is linked to lymphoma and other diseases; MEF2C has a correlation with congenital heart disease and myotonic dystrophy, among others; MEF2D is relevant to glioma, lung cancer, and myxoma, among other conditions [24].

**TABLE 1:** Summary of partial expression tissues and corresponding functions of MEF2.

Expression organization	Muscle [15]	Nerve [16, 17]	Blood vessel [18]	Tumor [19, 20]	Immune system [21, 22]	Bone [23]
**Function**	Promote differentiation of muscle cells and formation of muscle fibers, and affect muscle regeneration [15]	Affect the development and survival of neurons, limit the number of synapses in neurons, and may affect cognitive process, learning and memory formation [16, 17]	Influence on angiogenesis, differentiation, proliferation and apoptosis of vascular endothelial cells [18]	Promote or inhibit the development of tumor cells [19, 20]	Regulate the development and function of lymphocytes [21, 22]	Promote the development and function of bone related cells [23]

**TABLE 2:** MEF2 family members and their special roles in angiogenesis and fibrosis (related diseases adapted from [24]).

Type	MEF2A	MEF2B	MEF2C	MEF2D
Related diseases	Muscle degeneration Atherosclerosis	lymphoma	Congenital heart disease Myotonic dystrophy	Glioma lung cancer Myxoma
Roles in angiogenesis	Promote angiogenesis [25]	Unclear	Promote or inhibit angiogenesis (environment dependent) [26, 27]	Promote fibrosis [28]
Roles in fibrosis	Promote fibrosis [12]	Unclear	May promote fibrosis [29] (environmental dependent)	Promote fibrosis [30]

## 4 The Relationship Between Angiogenesis and Scar Formation

Wound healing has multiple stages, roughly divided into hemostasis, inflammation, proliferation, and remodeling [31]. The boundaries of these stages are not completely independent but partially overlap. The entire process is regulated by various regulatory factors such as cytokines and growth factors, ensuring that the microenvironment is always in a balanced and controllable state. Once it is intervened to exceed its regulatory range by external risk factors, it will lead to poor wound healing neoplasm and pathological scar formation [32].

### 4.1 Angiogenesis Is a Complex Process Involving Numerous Factors

Angiogenesis predominantly occurs during the proliferation and remodeling phases of the wound healing process, and is closely related to scar formation. The main process of proliferation involves the activation, proliferation, and other activities of related cells such as keratinocytes, fibroblasts, and ECs, the generation and replacement of temporary ECM, and the promotion of capillary growth by factors like hypoxia [33]. The remodeling period takes a long time (months to years), mainly including angiogenesis reduction, the replacement of different collagen (Type III collagen was gradually replaced by type I collagen), ECM remodeling, and the transformation of granulation tissue into mature scar [34]. This stage is crucial for wound healing, as it significantly influences the likelihood of subsequent scar formation. The initiation process of angiogenesis is related to cytokines such as VEGF and TGF‐β family members, while hypoxia stimulation is also necessary [35]. Then, angiogenesis‐related growth factors bind to surface receptors on ECs, initiating related signaling pathways and triggering the release of proteolytic enzymes, such as matrix metalloproteinases (MMPs), to dissolve the temporary skin basement membrane that formed before the remodeling stage. Subsequently, ECs migrate to the site that needs repair. As these migrating ECs mature, they form blood vessels, which transport oxygen and nutrients to meet the normal tissue consumption and the requirements for wound repair, thereby completing the process of tissue regeneration and primary blood vessel formation [36].

### 4.2 Imbalanced Regulation of Angiogenesis Leads to Abnormal Scar Formation

Angiogenesis is a dynamic process regulated by factors that promote and inhibit angiogenesis. Any intervention on these factors can disrupt the normal progress of the entire process, potentially leading to pathological healing due to obstruction of normal physiological processes. In the early phase of angiogenesis, a large number of capillaries are rapidly generated in the tissue [37], which can provide ample raw materials and oxygen for wound repair and timely eliminate useless and harmful substances produced by metabolism, ensuring successful physiological processes. However, the latest research shows that excessive vascular proliferation does not positively impact wound healing and may even promote abnormal scar formation [6, 7]. Part of the reason is that most of the neovascularization in the early stages of healing is often not mature enough to provide effective blood flow perfusion for the tissue [38]. Therefore, increasing the number of functional vascular networks and reducing non‐functional proliferative blood vessels may become important research directions for promoting wound healing and reducing scar formation. In addition, the healing of oral mucosa [39, 40] and fetal skin in the uterus [41, 42] is considered a remarkable scarless wound healing method. After healing, the appearance is almost indistinguishable from the normal skin before injury, with less angiogenesis and scarring development in the healing process than in the skin during the healing process. Essentially, it resembles a regenerative process, suggesting that inhibiting angiogenesis may facilitate wound healing and reduce scar formation during the wound healing process [43].

### 4.3 Anti‐Angiogenesis Has Widespread Applications in Scar Treatment

As a scar treatment method, anti‐angiogenesis has been widely studied and gradually integrated into clinical practice. Studies have shown that the application of vascular growth inhibitors such as VEGF antibodies can inhibit angiogenesis during wound healing, reducing the number of blood vessels without compromising the normal healing of the entire wound layer, thereby affecting scar formation [41]. Currently, an escalating number of clinical treatment methods for scars aim to inhibit angiogenesis, thereby achieving the therapeutic objective. Radiation therapy, often administered after keloid resection surgery, exhibits effective therapeutic and preventive outcomes against scar formation and recurrence [44]. This efficacy may stem from the high sensitivity of vascular ECs to radiation, enabling long‐term radiation therapy to diminish the count of hyperpermeable blood vessels in tissues [45]. Steroid hormones can inhibit angiogenesis by inducing vasoconstriction and reducing blood flow in scars [46]. Steroids also quickly alleviate itching and pain [47, 48] of scars, which may be the result of the lessened diffusion of inflammatory factors to surrounding tissues due to vasoconstriction. Vascular lasers, such as pulsed dye laser (PDL) and long pulse Neodymium‐doped Yttrium Aluminum Garnet (Nd: YAG) laser, target the hemoglobin in the blood vessels, thereby reducing their number by directly damaging them and alleviating scar symptoms [49, 50].

### 4.4 There Is an Intimate Relationship Between Angiogenesis and Pathological Scar Formation

Keloids and hypertrophic scars belong to pathological scars, and both are abnormal processes of fibrous hyperplasia. Currently, our knowledge of pathological scars is limited, and the causes of their occurrence are closely related to active neovascularization, excessive generation of collagen and other ECM proteins within tissues [51, 52]. Numerous studies have found that the vascular density in hypertrophic scar and keloids is significantly higher than that in normal skin tissue [6, 53]. However, it is not solely that vascular process that affects scar formation; there are other processes, including ECM, inflammation, macrophage phenotype transformation, cytokines, etc. [54]. The generation of ECM occurs throughout the entire process of injury healing, and its component varies at different stages of wound healing [55]. Collagen is the most common component, and excessive collagen synthesis or abnormal ECM accumulation can lead to scar formation in the late stage of wound healing [52].

## 5 The Mechanisms of MEF2 Involvement in Angiogenesis and Scar Formation

**FIGURE 1:**
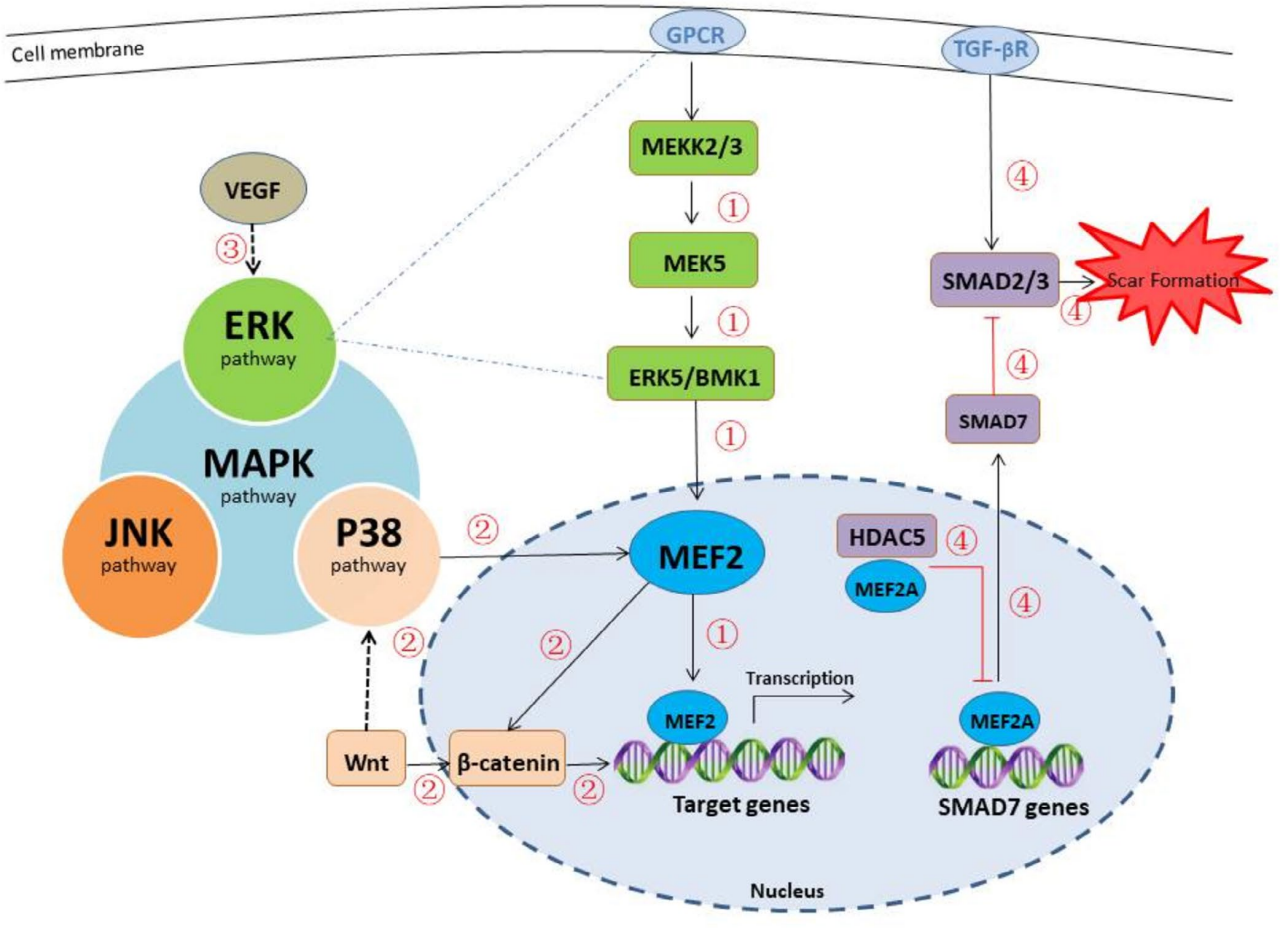
Possible mechanisms of MEF2 in angiogenesis and scar formation. The MAPK signaling pathway comprises multiple sub‐pathways, including the ERK, p38 MAPK, and JNK pathways. Among these, MEF2 is primarily associated with the ERK and p38 MAPK pathways. (1) The ERK pathway activates MEF2, which in turn stimulates downstream target genes involved in angiogenesis and contributes to the regulation of this process. (2) MEF2 participates in the crosstalk between the p38 MAPK pathway and the Wnt/β‐catenin signaling pathway, leading to the activation of downstream genes that promote the proliferation of vascular smooth muscle cells. Furthermore, the P38/MAPK pathway enhances inflammatory cell infiltration and vascular inflammatory responses through MEF2 activation, which also facilitates angiogenesis and scar formation. (3) VEGF upregulates MEF2C expression via the ERK pathway. Upon binding to its receptors, VEGF activates multiple signaling pathways, including the ERK and PI3K/Akt pathways, which collectively modulate angiogenesis. (4) MEF2A interacts with HDAC5 to form an HDAC5‐MEF2A complex. This complex inhibits MEF2‐mediated transcription of Smad7, thereby attenuating the antagonistic effect of Smad7 on Smad2/3. As a result, phosphorylation of Smad2/3 is enhanced, promoting Smad signal transduction and ultimately activating the TGF‐β/Smads pathway. This mechanism influences fibrosis and scar formation during wound healing. Abbreviation: ERK, extracellular regulated protein kinases; MEK5, mitogen‐activated protein kinase kinase 5; ERK5/BMK1, Extracellular signal‐regulated kinase 5/Big mitogen‐activated protein kinase 1.

Since the discovery of MEF2, research on it has been ongoing, and an increasing number of investigators have found that MEF2 is closely associated with angiogenesis and scar formation [56, 57]. Although there is currently no literature directly explaining the complete mechanism of MEF2's involvement in angiogenesis and scar formation, possible mechanisms can be summarized from some works. MEF2 may contribute to certain stages of wound healing, and its possible mechanisms mainly include indirectly affecting scar formation by participating in angiogenesis regulation and vascular inflammatory response, as well as directly stimulating fibroblasts and ECM [12, 58] (Figure 1).

### 5.1 MEF2 Participates in Angiogenesis Regulation and Vascular Inflammatory Response

The important role angiogenesis plays in scar formation has been previously introduced and will not be elaborated upon here. Inflammatory reactions arise during the early stages of wound healing, influencing subsequent angiogenesis and scar formation processes [54]. Chronic inflammation, a defining characteristic of pathological scars, readily fosters the development of high‐permeability and immature blood vessels during the initial phases of scar formation [59]. MEF2 indirectly affects scar formation by participating in angiogenesis regulation and vascular inflammatory response. This process involves numerous potential mechanisms, primarily the MAPK signaling pathway and its interaction with VEGF.

The MAPK signaling pathway is ubiquitous and encompasses several key types: The ERK pathway, the p38 MAPK pathway, and the JNK pathway [60]. Among the ERK protein kinases, there are five variants known as ERK1‐5. ERK5 is particularly associated with the activation of MEF2 transcription factors, playing a pivotal role in the survival and proliferation of vascular ECs. The intricate process of the ERK5 pathway involves a cascade reaction: MEKK2/3 activates MEK5, which then activates ERK5/BMK1, leading to the activation of MEF2. Subsequently, MEF2 stimulates downstream target genes related to angiogenesis, thereby influencing the survival and proliferation of ECs and the overall angiogenesis process [61]. VEGF, a well‐known growth factor [62, 63], enhances vascular permeability, promotes EC proliferation, and facilitates angiogenesis. Recent research by Li T et al. suggests that VEGF can upregulate MEF2C expression, and this upregulation is partially mediated through the ERK pathway [26]. Following the binding of VEGF to its receptors, various intracellular signaling pathways, including the ERK pathway and the PI3K/AKT pathway, are activated [64], further impacting the process of angiogenesis. P38, another member of the MAPK family, exhibits crosstalk with the Wnt/β‐catenin signaling pathways. Activation of the p38 MAPK pathway enables MEF2 to interact with β‐catenin, thereby enhancing β‐catenin accumulation. This, in turn, leads to the activation of downstream genes that promote the proliferation of vascular smooth muscle cells, potentially benefiting angiogenesis and scar formation [65]. Additionally, MEF2 proteins can negatively regulate the p38 MAPK pathway through a feedback mechanism [66]. The P38/MAPK signaling pathway also plays a role in promoting the infiltration of inflammatory cells, such as macrophages, by activating MEF2, thus mediating vascular inflammatory reactions and creating conditions for subsequent angiogenesis and scar formation [67].

### 5.2 MEF2 Directly Stimulates Fibroblasts and the ECM

Fibrosis is a pathological state of tissue characterized by abnormal deposition of ECM and excessive activation of fibroblasts [68]. It can affect various tissues and organs, leading to tissue hardening, structural abnormalities, and organ dysfunction [69, 70]. Scar formation is a specific example of fibrosis. Currently, TGF‐β is one of the most extensively studied molecules in scar formation, and it is centrally involved in the development of fibrosis and scar tissue. The TGF‐β/Smad signaling pathway enhances fibroblast reproduction and angiogenesis while inhibiting matrix metalloproteinase (MMP) activity. This dual action reduces ECM degradation, leading to excessive ECM deposition and increased scar formation [71]. MEF2A plays an important role in regulating the TGF‐β/Smad signaling pathway by mediating Smad7 transcription and inhibiting the pathway, thereby preventing the formation of hypertrophic scars. HDAC5 interacts with MEF2A, weakening the antagonistic effect of Smad7 on Smad2/3‐mediated signal transduction. By inhibiting MEF2‐mediated Smad7 transcription, HDAC5 further enhances Smad signal transduction by increasing Smad2/3 phosphorylation. Ultimately, this leads to the activation of the TGF‐β/Smads signaling pathway and its impact on fibrosis and scar formation during wound healing [12].

## 6 Discussion

Angiogenesis and fibrosis are involved in a wide range of physiological and pathological processes. Angiogenesis occurs primarily in special contexts such as early embryonic development, tissue injury, and tumor growth [72]. Fibrosis, as a key feature of scar formation, is observed not only in the skin [73] but also in internal organs [74], including the heart (e.g., post‐infarction scarring [75]), liver (e.g., cirrhosis due to hepatic fibrosis [76]), and lungs (e.g., pulmonary interstitial fibrosis [77]). Current research on the role of MEF2 in angiogenesis, fibrosis, and scarring has largely focused on specific organs (such as the heart and liver) and pathological conditions (including tumors and cardiovascular diseases) [78, 79], with relatively limited attention devoted to skin scar. Although direct evidence that selective inhibition of MEF2 reduces scar formation remains lacking, a growing number of studies indicate that MEF2 plays an important role in both angiogenesis [80, 81] and scar formation [12, 82].

Our article highlights the potential of MEF2 as a single target to concurrently modulate both vascularization and fibrosis. Compared to conventional single‐target approaches that influence each process independently, this strategy offers several advantages. It introduces novel mechanistic insight by proposing a new conceptual direction—whether MEF2 can simultaneously suppress angiogenesis and fibrosis merits further investigation. If confirmed, targeting MEF2 could disrupt the positive feedback loop between vascularization and fibrosis [83]. This approach may enhance clinical efficacy by improving the therapeutic outcome of scar treatment through synergistically addressing both key processes. Furthermore, compared to combination therapy involving separate agents for vascularization and fibrosis, a single MEF2‐targeted approach may reduce drug dosage, minimize side effects, avoid drug–drug interactions, and potentially offer a better safety profile.

This review also has several limitations: The currently limited available literature presents a challenge, as there are few publications directly addressing the role of MEF2 in scar formation. Most existing studies are based on in vitro models or animal experiments published some time ago [12, 82], which may subject our reference selection to potential bias and constrain our reliance on preclinical and animal‐derived evidence. Additionally, the context‐dependent functions of MEF2 subtypes introduce complexity, as their biological roles may vary—or even oppose one another—depending on tissue type, cellular microenvironment, timing, and other factors [84, 85]. For example, MEF2 has been reported to exert both promoting and suppressing effects on tumor development across different cancer types, and sometimes even within the same type [19, 20]. Although we haven't exhaustively summarized all such contextual variations, this complexity may conversely enhance the specificity of MEF2‐targeted therapies. Moving forward, more rigorous and well‐designed studies are needed to clarify the function of MEF2 in scar formation and to evaluate the therapeutic potential of MEF2 targeting for scar treatment.

Several experimental approaches could be employed to further investigate the direct effects of MEF2 on scar formation and angiogenesis. Initial analysis of clinical samples would involve collecting normal tissue and pathological scar tissues and conducting experiments such as immunofluorescence and immunohistochemistry to examine the expression levels, localization, and specificity of MEF2. For mechanistic studies, researchers could construct MEF2‐knockdown scar fibroblast cell lines using siRNA or Clustered Regularly Interspaced Short Palindromic Repeats (CRISPR) [86], then evaluate changes in fibrosis markers such as type I collagen, type III collagen, and α‐smooth muscle actin (α—SMA) in these cell lines and clinical samples through Western blot, qPCR, and related assays. Subsequently, apply transcriptome sequencing and protein interaction analyses to identify downstream target genes and binding partners of MEF2, thereby elucidating the molecular mechanisms through which MEF2 modulates angiogenesis and scar formation. Finally, validation in animal models: Establish conditional knockout or overexpression of MEF2 in mouse skin wound models [87], where locally administering regulators or inhibitors targeting upstream or downstream factors of MEF2 would allow assessment of alterations in angiogenesis and scar‐related indicators to evaluate the therapeutic effects of MEF2‐centered intervention on scar formation and vascularization.

## 7 Conclusion

MEF2 plays an important role in angiogenesis and scar formation. These processes, though complex and influenced by multiple factors, are interconnected, with MEF2 serving as a bridge between them. This article reviews relevant research and summarizes the relationship among MEF2, angiogenesis, and scar formation. It proposes that MEF2's participation in these processes may be facilitated through its regulation of angiogenesis and vascular inflammatory responses, as well as its direct stimulation of fibroblasts and the ECM. Various molecules and signaling pathways, including the MAPK signaling pathway, the TGF‐β/Smad signaling pathway, the PI3K/AKT signaling pathway, the Wnt/β‐Catenin signaling pathway, and VEGF, are also involved. These molecules and pathways are not independent but interact and influence each other. The processes of angiogenesis and scar formation are precisely regulated by this diversified and dynamic interplay involving MEF2. Future research on the MEF2 gene and its molecular mechanism may provide deeper insights into angiogenesis and scar formation, potentially offering new targets for the treatment of pathological scars.

## Author Contributions

Rui Tao finished the manuscript; Yajuan Song and Zhou Yu revised the manuscript; Tong Wang and Peng Guo designed the drawing part and provided the ideas; Yankun Guo and Yuye Cao modified the language; Yue Yin provided the framework and reviewed the manuscript.

## Ethics Statement

The authors confirm that the ethical policies of the journal, as noted on the journal's author guidelines page, have been adhered to. No ethical approval was required as this is a review article with no original research data.

## Conflicts of Interest

The authors declare no conflicts of interest.

## Data Availability

The data that support the findings of this study are available from the corresponding author upon reasonable request.
